# Supplementary material for: The neutrophil antimicrobial peptide cathelicidin promotes Th17 differentiation
Source: Nat Commun. 2021 Feb 24;12:1285. doi: 10.1038/s41467-021-21533-5 (PMC7904761; doi:10.1038/s41467-021-21533-5)
Supplement: Supplementary file 1 — Supplementary Information [file 41467_2021_21533_MOESM1_ESM.pdf]

Supplementary Figure 1 for Minns et al

The neutrophil antimicrobial peptide cathelicidin promotes Th17 differentiation

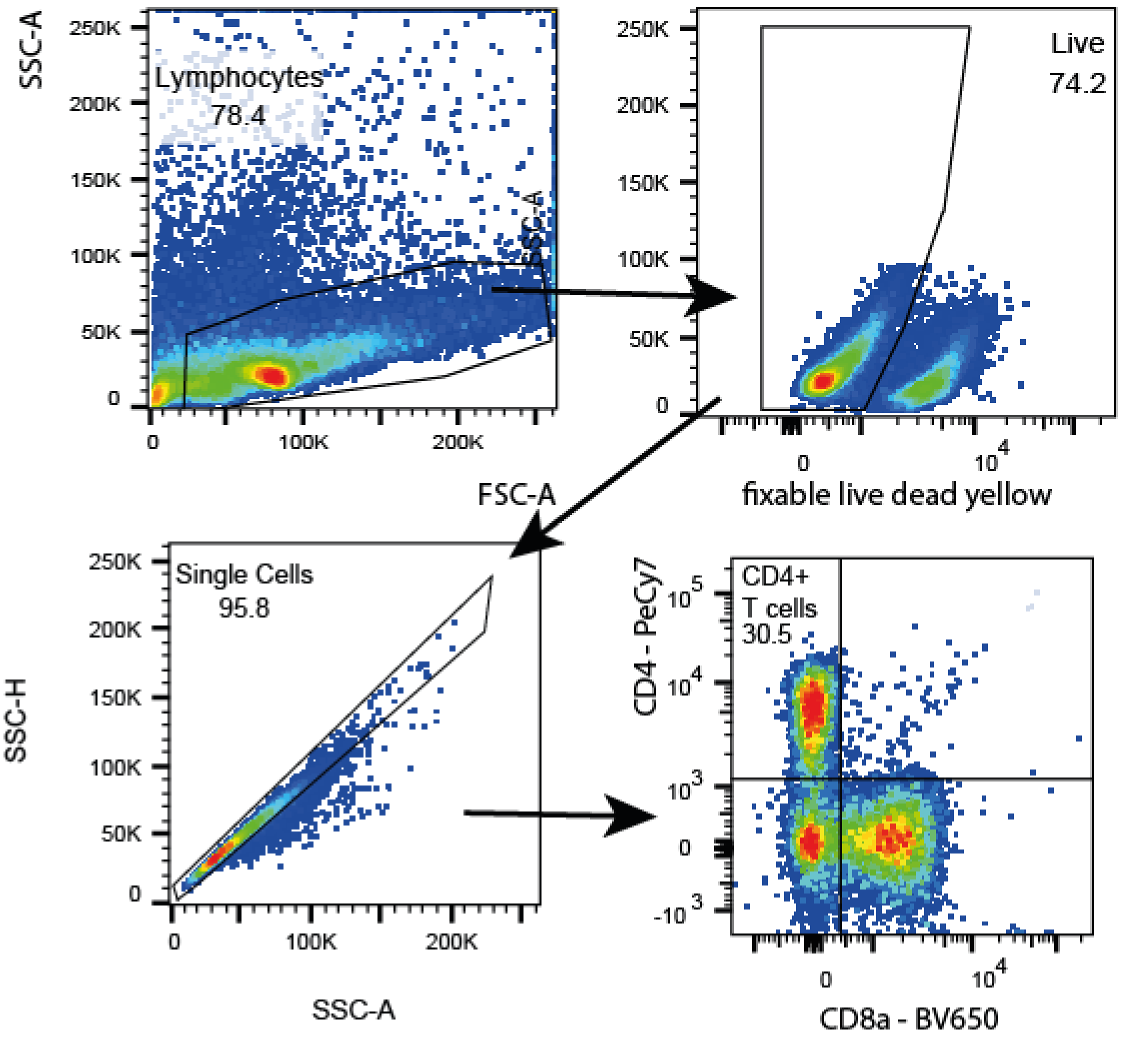

Supplementary Figure 1: Gating strategy for identification of CD4+ T cells.

Spleens and lymph nodes were removed from mice and single cell suspensions made. Following culture for up to 72 hours CD4+ T cells were identified by flow cytometry for further analysis according to this gating strategy.
