# Supplementary material for: The neutrophil antimicrobial peptide cathelicidin promotes Th17 differentiation
Source: Nat Commun. 2021 Feb 24;12:1285. doi: 10.1038/s41467-021-21533-5 (PMC7904761; doi:10.1038/s41467-021-21533-5)
Supplement: Supplementary file 3 — Reporting Summary [file 41467_2021_21533_MOESM3_ESM.pdf]

nature research

Corresponding author(s): Emily Gwyer Findlay

Last updated by author(s): Oct 16, 2020

Reporting Summary

Nature Research wishes to improve the reproducibility of the work that we publish. This form provides structure for consistency and transparency in reporting. For further information on Nature Research policies, see our Editorial Policies and the Editorial Policy Checklist.

Statistics

For all statistical analyses, confirm that the following items are present in the figure legend, table legend, main text, or Methods section.

n/a	Confirmed	
☐	☒	The exact sample size (*n*) for each experimental group/condition, given as a discrete number and unit of measurement
☐	☒	A statement on whether measurements were taken from distinct samples or whether the same sample was measured repeatedly
☐	☒	The statistical test(s) used AND whether they are one- or two-sided<br>*Only common tests should be described solely by name; describe more complex techniques in the Methods section.*
☒	☐	A description of all covariates tested
☐	☒	A description of any assumptions or corrections, such as tests of normality and adjustment for multiple comparisons
☐	☒	A full description of the statistical parameters including central tendency (e.g. means) or other basic estimates (e.g. regression coefficient) AND variation (e.g. standard deviation) or associated estimates of uncertainty (e.g. confidence intervals)
☐	☒	For null hypothesis testing, the test statistic (e.g. *F*, *t*, *r*) with confidence intervals, effect sizes, degrees of freedom and *P* value noted<br>*Give P values as exact values whenever suitable.*
☒	☐	For Bayesian analysis, information on the choice of priors and Markov chain Monte Carlo settings
☒	☐	For hierarchical and complex designs, identification of the appropriate level for tests and full reporting of outcomes
☒	☐	Estimates of effect sizes (e.g. Cohen's *d*, Pearson's *r*), indicating how they were calculated

Our web collection on statistics for biologists contains articles on many of the points above.

Software and code

Policy information about availability of computer code

Data collection	BD FACS Diva software version 9 was used for flow cytometry on a 5L BD Fortessa. Microscopy images were scaned on an Axioscan.Z instrument (Zeiss). Data was collected for Nanostring analysis by nCounter Advanced Analysis software.
Data analysis	Data was analysed with GraphPad Prism version 8 and FlowJo Version 10. nSolver version 4.0 was used to analyse the Nanostring data.

For manuscripts utilizing custom algorithms or software that are central to the research but not yet described in published literature, software must be made available to editors and reviewers. We strongly encourage code deposition in a community repository (e.g. GitHub). See the Nature Research guidelines for submitting code & software for further information.

Data

Policy information about availability of data

All manuscripts must include a data availability statement. This statement should provide the following information, where applicable:

- Accession codes, unique identifiers, or web links for publicly available datasets
- A list of figures that have associated raw data
- A description of any restrictions on data availability

The datasets generated during the current study are available from the corresponding author upon request. The authors declare that all data supporting the findings of this study are available within the paper. The Nanostring data have been uploaded to GEO, accession code GSE160757.

Field-specific reporting

Please select the one below that is the best fit for your research. If you are not sure, read the appropriate sections before making your selection.

- [x] Life sciences
- [ ] Behavioural & social sciences
- [ ] Ecological, evolutionary & environmental sciences

For a reference copy of the document with all sections, see nature.com/documents/nr-reporting-summary-flat.pdf

Life sciences study design

All studies must disclose on these points even when the disclosure is negative.

Sample size	Three mice or donor blood samples were performed for each experiment and then a power calculation performed to determine minimum sample size.
Data exclusions	No data were excluded from analysis.
Replication	All attempts at replication throughout the study were successful. Each individual in vitro culture of mouse or human cells was performed independently, so n values listed in figure legends equal independent experiments. In vivo experiments were performed at least twice with the exception of the influenza infection in Figure 8, which was performed once.
Randomization	Samples were not randomized. In each case one mouse spleen or blood sample was used and split into wells to be treated with cathelicidin or left untreated. Each such experiment was performed separately.
Blinding	The histological analyses were performed by an investigator who had not performed the upstream experiment and was not aware of the conditions. Influenza and vaccination experiments all involved one researcher (EGF) immunising or inoculating the mice and another harvesting tissues and running samples on the flow cytometer, such they did not know the groups until after the experiment.

Reporting for specific materials, systems and methods

We require information from authors about some types of materials, experimental systems and methods used in many studies. Here, indicate whether each material, system or method listed is relevant to your study. If you are not sure if a list item applies to your research, read the appropriate section before selecting a response.

Materials & experimental systems

n/a	Involved in the study	
☐	☒	Antibodies
☒	☐	Eukaryotic cell lines
☒	☐	Palaeontology and archaeology
☐	☒	Animals and other organisms
☐	☒	Human research participants
☒	☐	Clinical data
☒	☐	Dual use research of concern

Methods

n/a	Involved in the study	
☒	☐	ChIP-seq
☐	☒	Flow cytometry
☒	☐	MRI-based neuroimaging

Antibodies

Antibodies used	All details of antibodies used are listed in the Methods section.<br><br>Mouse:<br>CD4 (clone GK1.5, Biolegend, #100453, dilution 1:200); CD8 (53-6.7, BD Biosciences, #563786, 1:200); IFNγ (XMG1.2, Biolegend, 505825, 1:100); IL-17A (TC11-18H10.1, Biolegend, #506938, 1:100); IL-22 (POLY5164, Biolegend, #516411, 1:100); RORγT (B2D, eBiosciences, #12-6981-80, 1:100); TBET (4B10, Biolegend, #644805, 1:100); AHR (4MEJJ, eBiosciences, #25-5925-80, 1:100); pSTAT3 (LUVNKLA, eBioscience, #11-903-42, 1:100); pSMAD2/3 (o72-670, BD Biosciences, #562586, 1:100)<br><br>Human:<br>CD4 (clone OKT4, eBioscience #25-0048-42, 1:200), IL-17A (eBio64DEC17, eBioscience #12-7179-42, 1:100)), IL-17F (Poly5166, Biolegend #516604, 1:150).
Validation	All antibodies were bought from commercial suppliers and are validated by the vendor for the species and assay used in our study. Validation data is available on the vendors' websites. All antibodies were initially tested against unstained controls and dilution series performed to optimise. Subsequently, fluorescence minus one controls (FMO) were used to set gates and to determine any non-specificity of antibodies.

Animals and other organisms

Policy information about studies involving animals; ARRIVE guidelines recommended for reporting animal research

Laboratory animals	Mice strain C57BL/6J and fully-backcrossed cathelicidin knockout mice (Camptm1Rig) were used between 6 and 12 weeks of age. Both male and female mice were used and no differences were noted between the sexes. Mice were bred in specific-pathogen free conditions at the University of Edinburgh.
Wild animals	The study did not involve wild animals.
Field-collected samples	The study did not involve samples collected from the field.
Ethics oversight	Animal use was governed by Home Office licences PAF438439 and 70/8884 and the University of Edinburgh Animal Welfare and Ethical Review Board, which approved application for these licences and the general programme of work. All experiments were individually approved by University of Edinburgh Veterinary staff and all investigators trained in accordance with Home Office guidelines.

Note that full information on the approval of the study protocol must also be provided in the manuscript.

Human research participants

Policy information about studies involving human research participants

Population characteristics	Peripheral blood was taken from healthy donors recruited from University of Edinburgh staff and students. Both men and women were recruited, between 22 and 55 years of age. Exclusion criteria for being allowed to give blood were - infection with blood-borne diseases, previous or current intravenous drug use, anaemia, any blood clotting disorders or anticoagulant use, or regular use of steroids.
Recruitment	Healthy members of staff volunteered to be part of the blood donation system.<br>Students and staff were recruited by posters placed around the centre. If they volunteered to donate they were added to a centrally-managed list of donors, which was updated yearly. No inducements to donate were offered. Only students and staff in the Centre therefore joined the list, which may bias results to a younger cohort without illnesses which may make home-working more likely.
Ethics oversight	All procedures were in accordance with ethics AMREC 15-HV-013, project code 032. Experiments were overseen by the University of Edinburgh Centre for Inflammation Research Blood Resource Management Committee.

Note that full information on the approval of the study protocol must also be provided in the manuscript.

Flow Cytometry

Plots

Confirm that:

- [x] The axis labels state the marker and fluorochrome used (e.g. CD4-FITC).
- [x] The axis scales are clearly visible. Include numbers along axes only for bottom left plot of group (a 'group' is an analysis of identical markers).
- [x] All plots are contour plots with outliers or pseudocolor plots.
- [x] A numerical value for number of cells or percentage (with statistics) is provided.

Methodology

Sample preparation	Mouse spleens or lymph nodes were passed through a 100uM sieve before red blood cells were lysed. Cells were either plated as whole splenocytes or CD4+ T cells isolated (by cell sorting for live CD3+ CD4+ cells or using EasySep CD4+ isolation kit).<br>Blood collected from healthy human donors was treated with Ficoll Paque Plus to isolate mononuclear cells then CD4+ T cells isolated with EasySep CD4+ isolation or CD3+ isolation kits.
Instrument	Data was collected on a BD 5-laser Fortessa.
Software	Samples were collected with BD FACSDiva and analysed with FlowJo version 10.
Cell population abundance	CD4+ T cell purity was routinely over 95% following isolation.
Gating strategy	All experiments were gated first to identify lymphocytes, then live cells (using either DAPI exclusion or a fixable live-dead marker). Single cells were identified with FSC-A vs FSC-H gating then CD4+ T cells gated. From there cell surface markers or cytokines were analysed.

- [x] Tick this box to confirm that a figure exemplifying the gating strategy is provided in the Supplementary Information.
